# Analyzing the Freight Characteristics and Carbon Emission of Construction Waste Hauling Trucks: Big Data Analytics of Hong Kong

**DOI:** 10.3390/ijerph19042318

**Published:** 2022-02-17

**Authors:** Xiaoxuan Wei, Meng Ye, Liang Yuan, Wei Bi, Weisheng Lu

**Affiliations:** 1 School of Management Science and Real Estate, International Research Center for Sustainable Built Environment, Chongqing University, Chongqing 400045, China; 2 Department of Real Estate and Construction, The University of Hong Kong, Pokfulam Rd, Hong Kong, China; u3007096@connect.hku.hk (L.Y.); biwei.226@gmail.com (W.B.); wilsonlu@hku.hk (W.L.); 3 School of Economics and Management, Southwest Jiaotong University, Chengdu 610000, China; mengye@swjtu.edu.cn

**Keywords:** freight characteristics, construction waste hauling trucks, carbon emission, big data

## Abstract

Unlike their counterparts that are used for container or municipal solid waste hauling, or their peers of taxies and other commercial vehicles, construction waste hauling trucks (CWHTs) are heterogeneous in that they transport construction waste from construction sites to designated disposal facilities. Depending on the intensity of the construction activities, there are many CWHTs in operation, imposing massive impacts on a region’s transportation system and natural environment. However, such impacts have rarely been documented. This paper has analyzed CWHTs’ freight characteristics and their carbon emission by harnessing a big dataset of 112,942 construction waste transport trips in Hong Kong in May 2015. It has been observed that CWHTs generate 4544 daily trips with 307.64 tons CO_2_-eq emitted on working days, and 553 daily trips emitting 28.78 tons CO_2_-eq on non-working days. Freight carbon emission has been found to be related to the vehicle type, transporting weight, and trip length, while the trip length is the most influential metric to carbon emission. This research contributes to the understanding of freight characteristics by exploiting a valuable big dataset and providing important benchmarking metrics for monitoring the effectiveness of policy interventions related to construction waste transportation planning and carbon emission.

## 1. Introduction

By transporting goods, freights play a crucial role in a nation’s economy. This is particularly true with the increase in e-commerce, logistics, and supply chains around the globe. As a heterogeneous group for the logistics, construction waste has been one of the heaviest and most voluminous waste streams produced globally under the fast-economic development and intensive urban renewal [1,2,3]. In 2018, 600 million tons of construction waste were generated in the US, which is more than twice the amount of generated municipal solid waste [4]. In 2016, 66.2 million tons of non-hazardous construction waste were generated in the UK [5]. In 2019, the generation of construction waste in Hong Kong doubled since 2008, hitting nearly 18 million tons per annum [6]. Owing to its non-combustible nature, construction waste normally ends up in disposal facilities, such as public fills or landfills [7,8]. A large number of construction waste hauling trucks (CWHTs) have been mobilized to transport such construction waste to various disposal facilities [9].

CWHTs thus play a pivotal role in the entire construction waste management system [10]. However, little is known about how they perform. In general, the freight performance is regarded as the metrics that are associated with freight characteristics of the trucks, which can be investigated by several dimensions, such as transporting weight, vehicle type, trip length, trip time, and so forth, which can be exploited in order to better understand the current problems in transporting construction waste and support decision making. For example, the transporting weight for each trip is helpful in order to schedule trucks with suitable permitted gross vehicle weight. In reality, infrastructure systems are underutilized for most of the day, while overwhelmed during the peak hours [11]. A good knowledge of trip time can help planners to better cater for construction projects through improved design and use of facilities and infrastructure, and to arrange the transporting time. It is therefore essential to measure the CWHTs properly in order to provide a reference for the responsible parties (e.g., a shipment company, urban planners) to improve the efficiency of freight transportation. Without an understanding of the freight performance of CWHTs, the construction project may be delayed and cause economic loss.

In addition to the performance, freight transportation also causes significant negative environmental and social externalities for cities [12]. The transport sector has contributed to around 14% of total greenhouse gas emissions on average over the last ten years or so, with three-quarters from road transport [13]. Among the road transport emissions, 29.4% comes from freight movements [14]. As the backbone of urban road freight, CWHTs thus need to be closely scrutinized in terms of carbon emissions. It is commonly appreciated that freight carbon emission is related to freight performance. For example, trip length and transporting weight are important factors that influence the energy consumption for waste transportation, which can largely determine carbon emission in road transportation [15]. Moreover, similar to most hauling trucks, CWHTs are bulky and can easily cause road congestion, which is associated with frequent gear changing, resulting in increased carbon emission [16]. Investigating the relationship between CWHTs’ freight performance and carbon emission can help to formulate appropriate emission reduction policies.

This paper probes into construction waste hauling trucks (CWHTs) as a heterogeneous freight group and analyzes their characteristics and carbon emission, intending to provide a departure point for planning, optimization, and implementation of freight transportation and carbon emission reduction policies. It does so by focusing on Hong Kong as a highly built-up city and exploiting a big dataset therein. The remainder of the paper is organized as follows. Section 2 provides a critical literature review on freight performance metrics and freight carbon emission. Section 3 introduces the construction waste disposals in Hong Kong. Section 4 describes the data and methods. Section 5 elaborates the analyses and findings of the freight performance and carbon emission of CWHTs. The discussion is presented in Section 6, followed by Section 7, which draws the conclusion and policy implication.

## 2. Literature Review

### 2.1. Freight Performance Metrics

Freight performance metrics are regarded as analytical and interpretive tools to assist policy-makers in tackling problems associated with freight transportation and developing optimal freight strategies [17]. Although the metrics that are used are hardly consistent, they can be divided into the following two categories: vehicle-related and trip-related [18]. While the former describes the characteristics of the trucks, the latter depicts the variables that are affiliated with a trip. Khan and Machemehl (2017) reveal that these two categories of metrics can help us to understand efficient tour chain strategies [19]. Table 1 summarizes the two categories of freight performance metrics identified in the literature.

**Figure 1:**
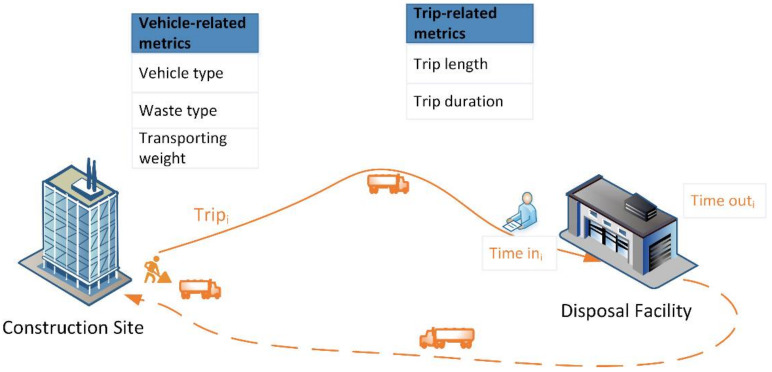
Freight performance metrics for construction waste hauling trucks.

Fourteen freight performance metrics are revealed in the literature with five vehicle-related metrics and nine trip-related metrics, which can describe the different freight activities. For example, Ehmke et al. (2012) examined the logistic trip duration to choose more reliable and efficient delivery tours [28]. Sulemana et al. (2019) examined the influencing factors of routing optimization for municipal solid waste collection with the respect to travel distance, travel time, and fuel consumption [29]. Comi et al. (2021) retrieved origin–destination (O–D) flows for light goods freight vehicles by delivery tour analysis [30]. Although the freight performance in terms of CWHTs is rarely explored, the literature can suggest the categories of freight performance metrics for this research (shown in Figure 1).

While the vehicle-related category includes vehicle type, waste type, and transporting weight, the trip-related category consists of trip length and trip duration. Vehicle type describes the CWHTs’ physical properties, including fuel type, permitted gross vehicle weight, unit energy consumption and so on. Waste type is the type of construction waste that is transported by CWHTs, which can be divided into inert waste (e.g., bricks, concrete, etc.) and non-inert waste (e.g., vegetation, timber, etc.) [31]. Transporting weight is the net weight of the construction waste transported by CWHTs. The trip length and trip duration are distance traveled and time consumed by the CWHTs from the construction site to the disposal facility, respectively. 

### 2.2. Freight Carbon Emission

Many studies have focused on freight carbon emission. By estimating freight carbon emission of logistics sectors in Hong Kong, To (2015) found that land transport is more environmentally friendly than sea and air transport [32]. Lee (2011) found that 12% of the supply chain carbon emission comes from the stage of goods distribution [33]. Tian et al. (2014) examined different regions’ freight carbon emission by various transport modes in China and found that highway freight plays a prominent role in the total carbon emission trajectory [34]. Some research proposes attention to alternative fuels with regards to their potential to reduce freight carbon emission, such as liquefied natural gas [35], or even all-electric trucks [36]. The freight carbon emission, however, is highly associated with a good freight transportation arrangement by considering the carbon emission efficiency [37], which requires an investigation of the freight performance metrics. For example, the choices of vehicle types are explored by Sim (2017), who found that the optimal weight of trucks will promote carbon emission reduction [38].

The following two kinds of methods have been applied for calculating freight carbon emission: “top-down” and “bottom-up” methods [39]. The “top-down” method apportions carbon emission to different sectors [40]. Ma et al. (2019), for example, adopted this method in order to decompose transportation carbon emission in different regions in China from 2007 to 2016, reporting that there are significant variations in the carbon emission of the eastern, central, and western regions of China [41]. Gilman et al. (2013) reported that carbon emission calculated by the “top-down” method is likely to be underestimated [42]. The “bottom-up” method, on the other hand, estimates carbon emission from fuel consumption, together with regional carbon emission factors. Hao et al. (2015) reflected that the major merit of this “bottom-up” method is its low data requirement to populate the model [43]. By using this method, Sun et al. (2017) calculated and obtained the carbon emission of urban traffic in Shanghai in 2014, using the number of vehicles, mileage, energy consumption structure, and carbon emission factors for different energy sources [44]. Lv et al. (2019) found that the total amount of freight carbon emission in China increased from 3.73 Mt in 1988 to 96.41 Mt in 2016 [45].

### 2.3. Freight Data

The freight performance is usually measured by manually collected data and the private sector database. The most popular methods of collecting data manually include questionnaires [35], roadside surveys [18,46], and enumerations [21]. The private sector database also contributes to the measurement of freight performance, such as the data source from the university [47] and second-by-second GPS data gathered by a private company [48]. While manual data collection needs abundant manpower and material resources [49], the private sector data are inaccessible to the public. The public high-quality database is thus desired for measuring freight performance.

The public database can be exploited due to the rapid development of the Internet, Internet of Things (IoTs), cloud computing, wireless sensor, and other technologies, within which the truck Global Positioning System (GPS) data are widely employed. The integration of GPS with the emission monitoring sensor system can measure real-time emission concentration of vehicles [50]. For example, Akter (2019) explores the freight characteristics with the truck GPS data [25], and the data can also be converted into a freight database in order to investigate the freight characteristics of hauling trucks [51]. However, there are several challenges associated with the application of GPS data. Only a handful of vehicles in the traffic network may be equipped to record data via GPS and the quality of GPS data may be interfered with by various factors, such as tall buildings, tunnels, highway overpasses, etc. Moreover, GPS data fail to provide detailed information on individual trucks, such as commodity type, weight carried, and the type of truck [47].

By overcoming the drawbacks of GPS data, administrative record data provide another public database for the freight performance measurement. For example, Yang et al. (2021) exploited a high-quality dataset provided by the Southern California Association of Governments in the U.S. in order to determine transportation volume [52]. Nevertheless, the availability of high-quality and detailed data regarding construction waste freight movements is typically poor, which limits the research on the freight performance of CWHTs [53]. Inspired by the success of administrative record data in measuring freight performance, this paper aims to probe into the freight performance and carbon emission of CWHTs based on “waste transaction” big data in Hong Kong.

## 3. Construction Waste Disposals in Hong Kong

This research is conducted in the context of Hong Kong, where a huge quantity of construction waste is generated and transported from construction sites to disposal facilities, and most importantly, these data are recorded by the government, presenting a large set of high-quality structured data for measuring the freight performance of CWHTs. In order to effectively manage construction waste, the Hong Kong government has enacted the Construction Waste Disposal Charging Scheme since 2006. It mandates that all construction waste must be transported to government waste reception facilities (i.e., public fills, sorting facilities, and landfills) [54]. The Hong Kong Environment Protection Department (HKEPD) records each of the trucks delivering construction waste to the waste reception facilities. HKEPD (2020) reports that approximately 17.62 million tons of construction waste are generated, with 1.44 million tons disposed into landfills and 16.18 million tons received at public fills [6].

Table 2 summarizes construction waste disposal facilities and the types of accepted construction waste in Hong Kong. Consequently, different types of construction waste are expected to be transported to the designed disposal facilities. For example, inert materials, comprising mainly sand, bricks, and concrete, should be transported to public fills [55]. Based on the disposal facilities, the freight trips of CWHTs in Hong Kong can be categorized into the following three types: trips to public fills (TPF), trips to sorting facilities (TSF), and trips to landfill facilities (TLF).

**Figure 2:**
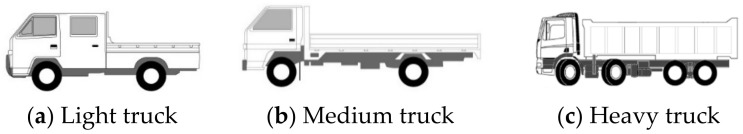
Illustrative schematic of truck classifications in Hong Kong.

Construction waste hauling trucks (CWHTs) for transporting construction waste must be registered at the HKEPD, with their plate numbers and permitted gross vehicle weight (PGVW) recorded. As the maximum gross weight assigned or determined concerning the vehicles, PGVW is always regarded as the basis for classifying CWHTs. By referring to the Hong Kong Transport Department vehicle class definition system, the registered CWHTs can be categorized into light trucks (0 < PGVW ≤ 5.5), medium trucks (5.5 < PGVW ≤ 24), and heavy trucks (24 < PGVW ≤ 38), examples of which are shown in Figure 2.

## 4. Data and Method

### 4.1. Data Description

**Figure 3:**
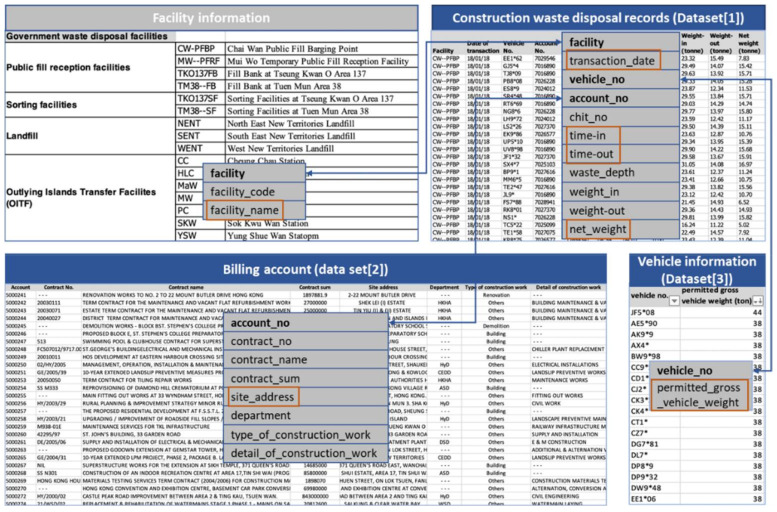
Three datasets and their interdependency.

Data used for this research were obtained from the HKEPD, consisting of three interdependent datasets, including datasets of construction waste disposal records (Dataset 1), billing account (Dataset 2), and vehicle information (Dataset 3). An excerpt of the three datasets and their interdependency can be understood from Figure 3.

Dataset 1 describes construction waste disposal records by the waste disposal facilities, which records information on every load of construction waste, including the facility name, transaction date, vehicle number, account number, time-in, time-out, and net weight, etc. Detailed information on these parameters is available in Table 3. This leads to a database of more than one million transaction records in a single year, which is considered to be a full coverage of the waste generated from all of the construction sites in Hong Kong. This “transaction records” dataset is composed of more than 12.7 million construction waste transaction records, from 2011 to 2019.

Dataset 2 describes billing account information. Before using disposal facilities, the responsible party (e.g., the main contractor if the contract exceeds HKD 1 million, or an individual, such as a vehicle owner or a small contractor if the contract values less than HKD 1 million) is mandated to register a billing account in the HKEPD. Dataset 2 thus describes basic information of all of the construction projects including the account number, contract name, contract sum, site address, and the type of construction work, etc. Particularly, the site address in Dataset 2 is critical to identify the origins of construction waste hauling trucks fleet trips, and this dataset is linked with Dataset 1 through account number. Dataset 3 describes CWHTs with vehicle numbers and permitted gross vehicle weight (PGVW) recorded. There are more than 10,000 CWHTs traced.

The datasets cover more than 6 million transporting records of construction waste over the past five years and the number is increasing at the rate of 4000 records per day. A broad range of elements is involved in the datasets. Due to the three Vs (volume, velocity, and variety) characteristics, the dataset is qualified as big data. By mining the big data, it is anticipated that the freight performance of CWHTs, as well as the related carbon emission in Hong Kong, can be measured.

### 4.2. Research Design

**Figure 4:**
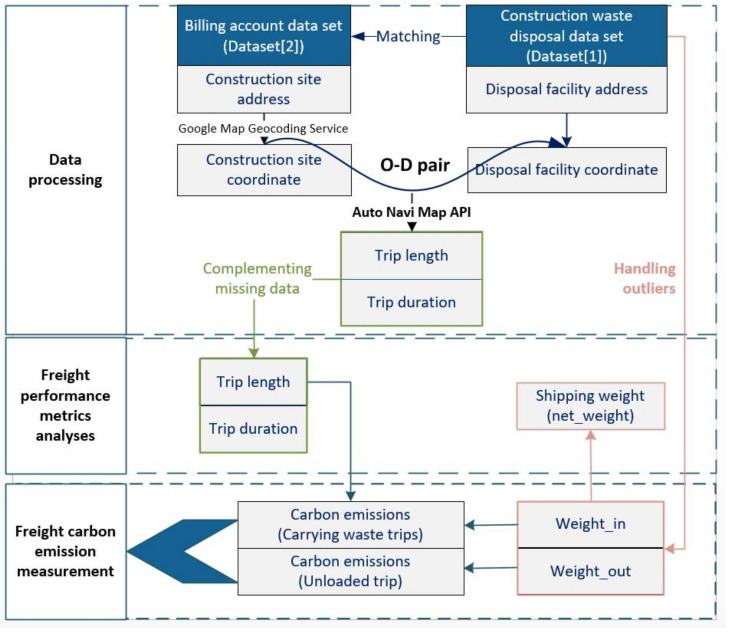
Research design.

To facilitate the understanding of our research design, Figure 4 illustrates the research framework, which includes data processing, freight performance metrics analyses, and freight carbon emission measurement.

#### 4.2.1. Data Processing

Two tasks were involved in the data processing. The first task was to match records and determine the origin–destination (O–D) pairs. A random month (May 2015) was chosen for this research with 113,025 transporting records shown in Dataset 1. The “facility” in Dataset 1 records the disposal facilities, which are the destinations of CWHTs. By referring to the account number and vehicle number, the records can be linked to “site address” in Dataset 2, showing where CWHTs load construction waste. Further to the names of O–D pairs, the geographical coordinates need to be further obtained for estimating the trip length and duration.

By engaging Google Maps Geocoding Service, the longitude and latitude data for each construction site (the origin) and disposal facility (the destination) are geocoded. Based on this, the trip length and duration can be extracted by applying the Auto Navi Map Application Programming Interface (API) in Python. This step follows the following two assumptions: (a) the driving trajectory is along with the shortest route for each O–D pair; (b) trip durations for the same O–D pair are static without considering traffic congestion (e.g., on-peak and off-peak period) or infrastructure changes (e.g., ad hoc diversion). In this regard, each trip length is assumed to be the shortest travel distance from the construction site to the disposal facility, and the corresponding trip duration is calculated regardless of the actual traffic situation but with the free traffic flow state.

The other task was data cleansing. A total of 83 (0.07% of data) transporting records were recognized as outliers by considering “weight_in” or “weight_out” (e.g., “weight_in” recorded with 3000.7 kg). These outliers were removed to ensure the appropriateness of further analyses. For the cases where site addresses were missing, the trip length and duration were complemented by using the mean value of trip length and duration of the corresponding freight trip type as well. This data processing was implemented in Matlab, which is an open-source statistical analytical software. Consequently, 112,942 effective transporting records in May 2015 were finalized for further analyses in this study.

#### 4.2.2. Freight Performance Metrics Analyses

The vehicle type, one of the vehicle-related freight performance metrics, was analyzed and summarized according to the freight trip types. Three kinds of trip-related freight performance metrics were further analyzed, including transporting weight, trip length, and trip duration. Transporting weight can be denoted by the “net_weight”, which was calculated by using the differences between “weight_in” and “weight_out”. The trip-related freight performance metrics were further analyzed statistically by showing histograms and box charts based on the vehicle types and freight trip types.

#### 4.2.3. Freight Carbon Emission Measurement

Freight carbon emission was measured by multiplying the energy consumption by the carbon emission factor [56]. The first step is to estimate energy consumption, which is determined by multiplying the trip length and the according average energy consumption. The Electrical and Mechanical Services Department in Hong Kong has provided the reference for the average energy consumption based on the gross vehicle weight (GVW, including the weight of the vehicle and the construction waste), shown in Table 4. For example, if GVW is less than 2.5 tons, the average energy consumption for the freight is 10.2 L/100 km. The effectiveness of the data has been widely demonstrated for calculating carbon emission in previous studies. For example, Bi et al. [57] used average energy consumption based on gross vehicle weight to calculate the carbon emission of the construction waste transportation.

The daily trip-chaining patterns of CWHTs can be investigated and summarized with the finding that most (more than 60%) trips follow a single site-single facility pattern. CWHTs may take business from other nearby sites as well, but this would not cause significant differences. To simplify, we assume that each CWHT has a round trip between the construction site and the disposal facility, with one carrying waste and the other unloaded. Therefore, the GVWs for the fourth and return trips are different. Following the bottom-up approach for carbon emission calculation, the energy consumption for each CWHT can be estimated by utilizing the following equation:

(1) $$E_{i}=e_{i}\times TL_{i}+e_{i}{}^{\prime }\times TL_{i}$$

where $E_{i}$ represents the energy consumption for the *i*th trip; $e_{i}$ represents the average energy consumption based on GVW of the *i*th carrying-waste trip; $e_{i}{}^{\prime }\;$ represents the average energy consumption based on GVW of the *i*th unloaded trip; and $TL_{i}$ denotes the on-way trip length for the *i*th trip.

The energy consumption can be further calculated into the freight carbon emission based on the carbon emission factor, which varies according to the carbon emission types. By referring to the HKEPD (2010), the three main types of carbon emission contain the emission of carbon dioxide (CO_2_), methane (CH_4_), and nitrous oxide (N_2_O), which can be examined by carbon dioxide equivalents (CO_2_-eq) [58]. Different types of carbon emission may lead to global warming to a different extent, which can be called the global warming potential (GWP). The freight carbon emission for each type of carbon emission can be calculated following Equation (2), as follows:

(2) $$C_{ij}=\underset{i}{\sum }E_{i}\times F_{j}\times GWP_{j}$$

where $C_{ij}$ represents the carbon emission for each trip and each type (in tons CO_2_-eq); $E_{i}$ represents the energy consumption for the *i*th trip, $F_{j}$ represents carbon emission factor for *j*th type of carbon emission, $GWP_{j}$ represents global warming potential for *j*th type of carbon emission, *i* represents trips, and *j* stands for CO_2_, CH_4_, and N_2_O. According to the Carbon Reduction Certificate created by Hong Kong Green Organization Certification (HKGOC, 2021), carbon emission factors for different types of CWHTs can be obtained (See Table 5) [59]. Additionally, IPCC (2006) defined the GWP for CO_2_, CH_4_, and N_2_O to be 1, 21, and 310, respectively [60]. As energy consumption is the same for the three types of carbon emission, the total freight carbon emission can be obtained by the following equation:

(3) $$C_{i}=\underset{i}{\sum }E_{i}\times \sum_{j}(F_{j}\times GWP_{j})$$

where $C_{i}$ represents the carbon emission for each trip (in tons CO_2_-eq); $\sum_{j}(F_{j}\times GWP_{j})$ denotes the total transferring factor, which is determined by the vehicle types. As CWHTs in Hong Kong are diesel, the transferring factors for light, medium, and heavy CWHTs were calculated to be 2.78, 2.84, and 2.84, respectively, the detailed process can be seen in Table 5.

## 5. Data Analyses, Results, and Findings

### 5.1. Freight Performance

#### 5.1.1. Vehicle Type

Table 6 presents the different trucks that are used for freight trip types. The heavy trucks are mostly used for carrying waste, accounting for 56.05% of the total trips, particularly for the trips to public fills, i.e., 62.68% of the trips to TKO137FB and 68.98% of the trips to TM38—FB. The medium trucks are also frequently used (43.64%), while only 0.32% of trips are conducted by the light trucks. Most of the trips to landfill facilities (i.e., 87.97% to NENT and 72.87% to SENT), and the majority of the trips to sorting facilities (i.e., 90.97% to TKO137SF, and 83.72% to TM38—SF), use the medium trucks. This can imply that the amount of construction waste transported to public fills is large enough for the heavy trucks to load, while for trips to landfills and sorting facilities, medium trucks are enough. 

#### 5.1.2. Transporting Weight

**Figure 5:**
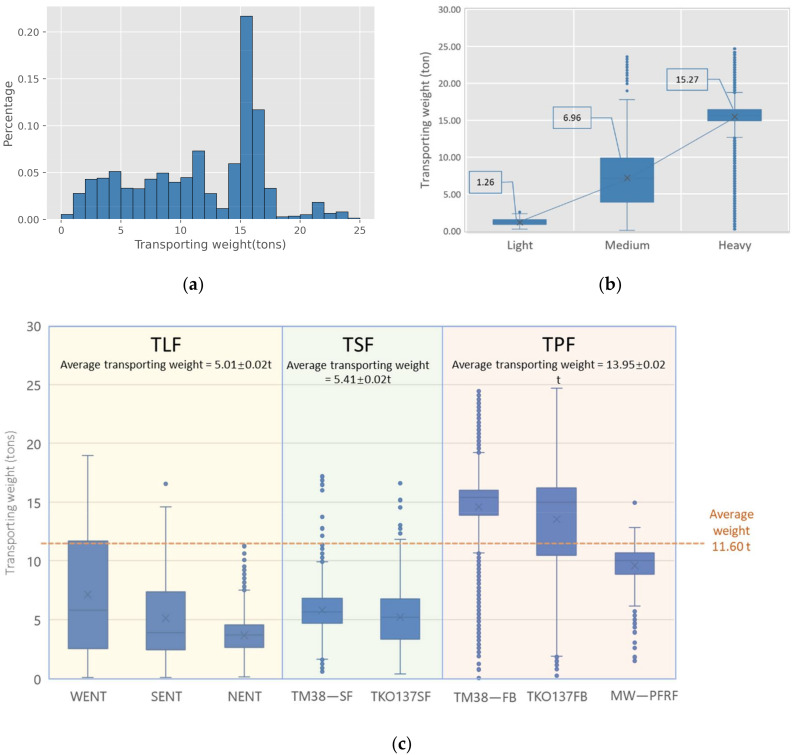
Transporting weight of CWHTs: (**a**) transporting weight histograms of CWHTs; (**b**) transporting weight distribution by vehicle types; (**c**) transporting weight distribution by freight trip types. Notes: Average transporting weight in (**c**) is presented as mean value ± SEM (standard error of the mean).

Transporting weight is considered to be a vital freight performance metric. Figure 5 illustrates the transporting weight of CWHTs in May 2015. It is noted from Figure 5a that the transporting weight for each trip ranges from 0 to 25 tons, with 15 to 17 tons occupying the highest probability. Figure 5b reports that the average transporting weight by the light trucks is 1.26 tons, while it is 6.96 tons for the medium trucks, and 15.27 tons for the heavy trucks. Drawn from the “weight_out” data from Dataset 1, we can get the weight of the empty trucks. The average weights for the light, medium, and heavy trucks are 3.5, 13.4, and 14.5 tons, respectively. By adding the transporting weight and the truck weights, the average gross vehicle weights for the light, medium, and heavy trucks are 4.76, 20.36, and 29.77 tons, respectively. Compared with the maximum PGVW for each truck type, say, 5.5, 24, and 38 tons, it has been found that on average, the gross vehicle weights can reach 86.5% (4.76/5.5) of the maximum PGVW for the light trucks, 84.8% (20.36/24) for the medium trucks, and 78.3% (29.77/38) for the heavy trucks. This indicates a relatively high loading ratio for all of the truck types and that the medium trucks have higher utilization efficiency than the heavy trucks. Figure 5c shows the transporting weights for the different trip types. The average transporting weights for TLF, TSF, and TPF are 5.01 tons, 5.41 tons, and 13.95 tons, respectively. Compared with the trips to the landfill and sorting facilities, the demands of transporting entirely inert construction waste to public fills are larger, evidenced by the high transporting weights per trip.

#### 5.1.3. Trip Length

**Figure 6:**
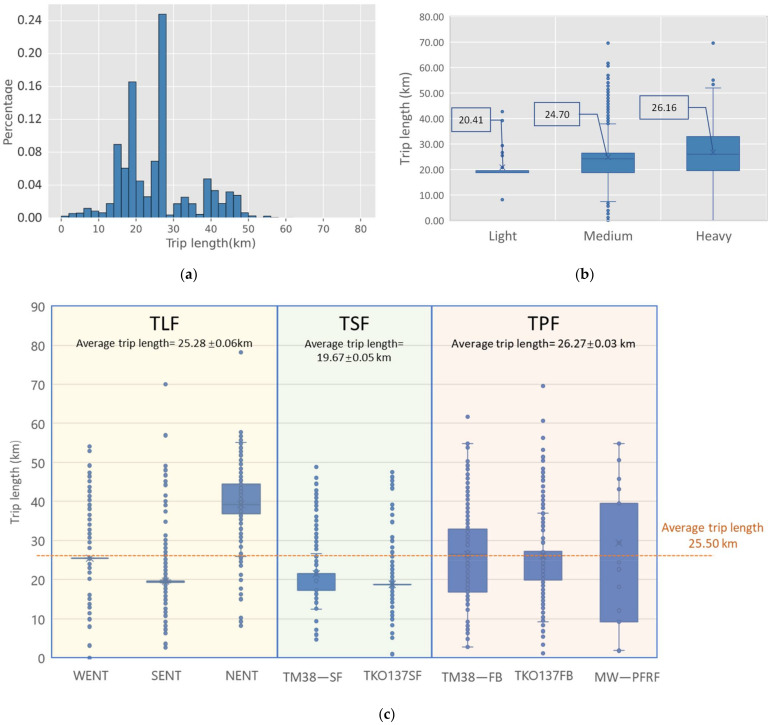
Trip length of CWHTs: (**a**) trip length histograms of CWHTs; (**b**) trip length distribution by vehicle types; (**c**) trip length distribution by freight trip types. Notes: Average trip length in (**c**) is presented as mean ± SEM (standard error of the mean).

Figure 6 presents the trip length distributions of CWHTs. The trip length is about 26 km on average, with the longest trip length being about 80 km. Figure 6a presents a considerable proportion of the trip length within 30 km, with 24% of the trip length 26–28 km and 16% of 18–20 km, indicating that short or medium distance transportation is preferred, and it is likely that drivers will choose the nearby disposal facilities. It can be shown in Figure 6b that the average trip length for each vehicle type is almost the same, indicating that the vehicle type would not affect the trip length of CWHTs. Figure 6c shows the trip length for the different freight trip types. The average trip length for TSF is 19.67 km, which is shorter than that of the overall average trip length, while trip lengths for TPF are distributed around the average level. This indicates that most of the drivers are supposed to choose a disposal facility with a suitable trip length, which is to say, the trip length would be an important factor to be considered for the construction waste freight transportation.

#### 5.1.4. Trip Duration

**Figure 7:**
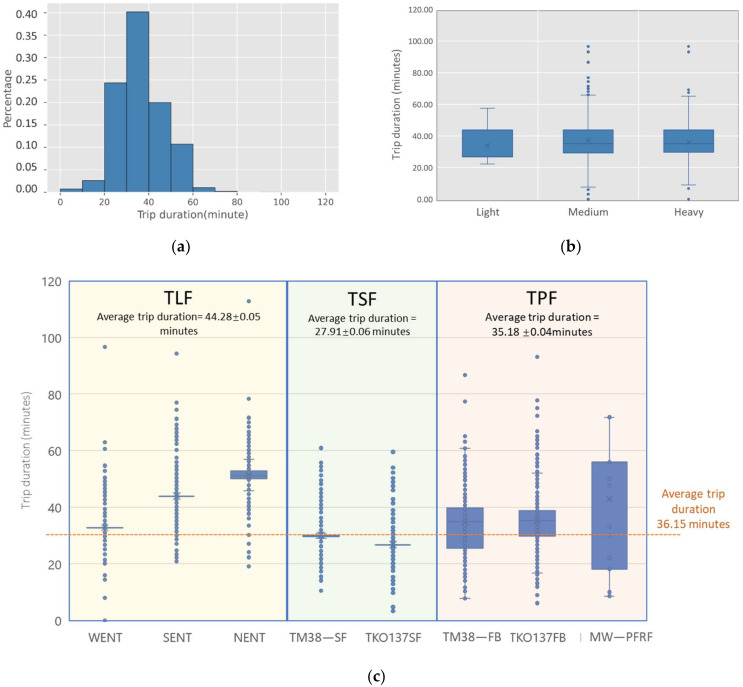
Trip duration of CWHTs: (**a**) trip duration histograms of CWHTs; (**b**) trip duration distribution by vehicle types; (**c**) trip duration distribution by freight trip types. Notes: Average trip duration in (**c**) is presented as mean ± SEM (standard error of the mean).

The trip duration is highly related to the trip length. The average trip duration is 36.15 min, with the longest duration of 120 min. Figure 7a shows that most of the trip durations for CWHTs range from 30 to 40 min, accounting for 40% of the total trips. Similar to the trip length, Figure 7b shows that the average trip duration for each type of CWHT show no obvious differences, indicating no significant effects of the vehicle type on the trip duration. Figure 7c reveals that TLF takes the longest time on average with a mean of 44.28 min. While TSF takes 27.91 min, a shorter time than the overall average trip duration, trip durations for TPF are distributed around the average level, with the average duration of TPF 35.18 min.

### 5.2. Carbon Emission Performance

**Figure 8:**
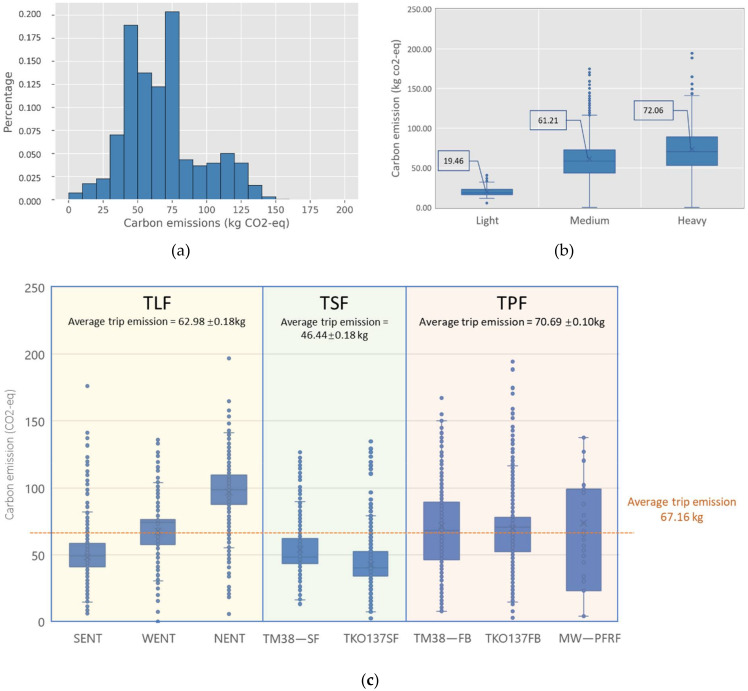
Carbon emission of CWHTs: (**a**) carbon emission histograms of CWHTs; (**b**) carbon emission distribution by vehicle types; (**c**) carbon emission distribution by freight trip types. Notes: Average trip emission in (**c**) is presented as mean ± SEM (standard error of the mean).

Referring to Equations (1)–(3), the carbon emission generated from the construction waste transportation in May 2015 can be calculated and shown in Figure 8. On average, construction waste transportation contributes 67.16 kg CO_2_-eq emission for each trip. Most of the trips emit 40 to 80 kg CO_2_-eq, as shown in Figure 8a. Referring to Figure 8b, the average construction waste carbon emission produced by the light, medium, and heavy trucks is 19.46, 61.21, and 72.06 kg CO_2_-eq, respectively, revealing that regardless of other factors, such as trip length, the heavier trucks seem to generate more carbon emission. Figure 8c shows that the average trip carbon emission of TLF, TSF, and TPF were 62.98, 46.44, and 70.69 kg CO_2_-eq, respectively. In particular, the trips to “NENT” generate the most carbon emission, with an average of 96.74 kg CO_2_-eq, due to its longest trip length. By considering the transporting weight and trip length, the trips to “NENT” are characterized with the lowest transporting weight but longest trip length, resulting in the lowest efficiency.

In order to better illustrate the carbon emission efficiency of construction waste transportation, we calculate the carbon emission for transporting one-ton construction waste, as shown in Table 7. In general, the larger the value of carbon emission for transporting one-ton construction waste, the lower the carbon emission efficiency. It shows that TPF has the highest carbon emission efficiency (5.07 kg CO_2_-eq/ton waste), followed by TSF (8.58 kg CO_2_-eq/ton waste) and TLF has the lowest (12.56 kg CO_2_-eq/ton waste). HKGOC (2021) shows that the carbon emission calculation needs to consider the vehicle type, the transporting weight, and the trip length of freight trips by CWHTs [59]. The TLF has low carbon emission efficiency due to the following two reasons: (1) the long trip length to “NENT”, one of the landfill facilities; (2) the majority of TLF uses the medium and heavy trucks but just loads an average of 5.01 tons of transporting weights, leading to more trips and, thus, longer total trip length.

### 5.3. Daily Freight Performance and Carbon Emission

Further to the analysis for one month, daily freight performance and carbon emission are explored in order to present the brief picture of construction waste freight transportation in one time dimension (a single day) and to illustrate the relationships among the metrics. For the daily analysis, working days and non-working days are analyzed separately. Table 8 shows the detailed daily freight performance and carbon emission for working days and non-working days.

Table 8 shows that among the total of 4544 trips made per working day, the 3390 TPF accounts for the largest proportion (74%). It is followed by TLF, which accounts for 17% of the total daily trips and TSF accounts for only 9%. Accordingly, a total of 53,110.52 tons of construction waste are transported per working day, and about 90% of which are disposed into the public fills. The result implies that inert construction waste transportation is the most significant contributor to the freight movements on working days. Comparably, the freight performance of CWHTs presents a significantly different situation on non-working days. Fewer trips are generated on non-working days, with an average of 553 daily trips and 5126.75 tons of daily transporting weights. The majority (46%) of freight trips are TLF on non-working days, followed by TPF (37%). This result is in line with the fact that two public fills (i.e., “MW—PFRF” and “TKO137FB”) are closed during non-working days.

The average daily carbon emission for the construction waste transportation by CWHTs is also shown in Table 8, which are 307.64 and 28.78 tons CO_2_-eq on working days and non-working days, respectively. On working days, TPF is the largest carbon emission contributor, accounting for 78%, followed by TLF (16%) and TSF (6%). During non-working days, carbon emissions produced by TLF, TSF, and TPF were 14.49, 4.05, and 10.24 tons CO_2_-eq, respectively. It has been found that the proportion of carbon emission for different freight trip types is highly consistent with the trip lengths, showing a highly related relationship between the trip length and carbon emission.

**Figure 9:**
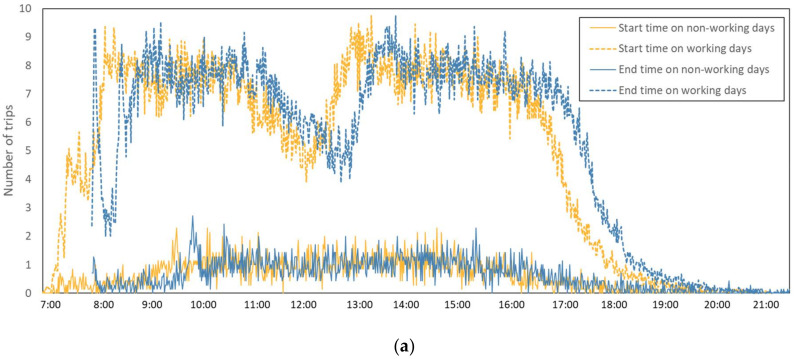

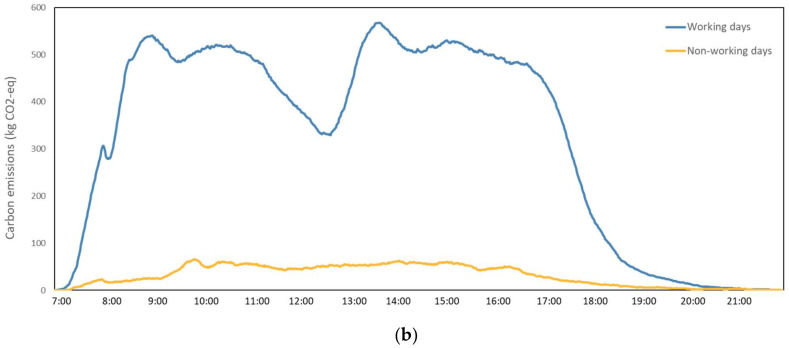
Time of day profiles of freight tips and carbon emission in Hong Kong: (**a**) Number of trips; (**b**) Carbon emission. Note: “end time” was described as the “time_in” in the Dataset 1; “start time” was calculated as “end_time” minus “trip duration”.

The analyses of daily performance and carbon emission can be further examined by time-of-day profiles. As plotted in Figure 9a, the line of the start time for the freight trips shows that freight trips of CWHTs start at 7:00 a.m. and continue until 10:00 p.m. Most of the CWHTs start freight trips for construction waste during 8:00–11:00 a.m. and 1:30–4:00 p.m., which seems to aggravate traffic congestion during the morning peak hours (7:00–9:00 a.m.). It is suggested to select the trip start time that would avoid the morning peak hours. The end time of the freight trips represents the time to enter the disposal facilities, and the line in Figure 9a shows the busy time of the disposal facilities during 8:30–11:30 a.m. and 2:00–5:00 p.m. on working days. During those periods, the disposal facilities are suggested to improve efficiency in order to deal with the large amount of the CWHTs. On the contrary, the lines of start time and end time for CWHTs show a different view on non-working days. There are no obvious busy hours for CWHTs and the CWHTs transport construction waste during 10:00–16:00 on non-working days. Accordingly, the average freight carbon emission on both working and non-working days is shown by minutes in Figure 9b. We can see that the freight carbon emission is intensive during 8:30–11:00 a.m. and 1:30–5:00 p.m. on working days, but there is no obvious intensive period on non-working days. By dividing trip duration by trip length, it is interesting to find that it takes a longer time (2.69 h/100 km) on non-working days compared to that (2.35 h/100 km) on working days (shown in Table 8), although the traffic congestion is not that serious on non-working days.

## 6. Discussion

This research takes advantage of an existing big dataset of construction waste transportation in order to understand the characteristics of freight performance and carbon emission of CWHTs in Hong Kong. When comparing the vehicles that are used for different types of trips, it was found that heavy trucks are mostly used for TPF, while other types of trips prefer to use medium trucks. This may be because of the high demands of transporting entirely inert waste to public fills, evidenced by the fact that the transporting weights of TPF occupy 89% of the total transporting weights per working day. The choices of vehicle types are supposed to be determined by the demands of transporting construction waste.

The trip length from construction sites to different facilities was calculated around 26 km on average, with a considerable proportion of the trip length within 30 km. This indicates that short- or medium-distance transportation is preferred by the CWHT drivers. By comparing the number of trips to the different disposal facilities and their locations, it can be found that facilities in the closer area, with a suitable trip length, are supposed to be chosen. The average trip length for each vehicle type is without obvious differences, indicating that the vehicle type would not affect the trip length.

By referring to the carbon emission calculation method provided by HKGOC (2021), the vehicle type, the transporting weight, and the trip length are supposed to affect the carbon emission of CWHTs freight transportation [59]. TPF uses heavy trucks with higher transporting weights, which are supposed to take more fuel and emit more carbon emission. However, the total trip length for transporting entirely inert waste to public fills would be reduced since the times of freight transportation would be less. Evidenced by the high carbon emission efficiency for TPF, trip length is supposed to be more important, compared to the transporting weight and the vehicle type, when calculating carbon emission. It is thus suggested that the trip length (trip length per trip as well as the total trip length) is a considerable metric to well coordinate freight transportation in terms of carbon emission. As opined by Yazdani (2021), carbon emission reduction can be achieved by optimizing the routing between the construction site and the disposal facilities.

The choices of vehicle types also matter to the carbon emission of CWHTs freight transportation. A relatively high loading ratio was found for all of the truck types, reaching around 80% on average. However, some types of trips still suffer a low loading ratio, for example, the majority of TLF uses the medium or heavy trucks but just loads an average of 5.01 tons of transporting weights. This leads to more trips and, thus, more carbon emission. It is thus suggested to choose suitable vehicle types based on the consideration of carbon emission. By involving the construction waste disposal charging scheme, the Hong Kong government encourages on-site waste sorting [61]. For example, the public fills charge HKD 71 per ton to accept entirely inert construction waste, while sorting facilities charge HKD 175 per ton for more than 50%, by weight, of inert construction waste [58]. Accordingly, the demands of transporting different types of construction waste to different facilities need to be considered when choosing vehicle types.

The time-of-day profiles provide the start time of freight trips for CWHTs during 8:00–11:00 a.m. and 1:30–4:00 p.m. on working days, aggravating the traffic congestion during the morning peak hours (7:00–9:00 a.m.), and the end time of freight trips showing the busy time of disposal facilities during 8:30–11:00 a.m. and 1:30–5:00 p.m. on working days. On the contrary, there is no obvious intensive period on non-working days for all of the facilities. An interesting finding shows that the trips on non-working days take a longer time than those on working days, by calculating trip duration/trip length. This may be because the demands for transporting construction waste on working days are larger than those on non-working days, drivers are motivated by the transporting behavior. A dynamic pricing system can be proposed with different transporting fees in order to disperse the freight transportation of CWHTs, with consideration of time constraint or loading ratio. For example, higher transporting fees can be paid at off-peak hours, or non-working days, by considering the road network congestion in order to improve transportation efficiency and to reduce carbon emission caused by traffic congestions [62]; additionally, a low-load fee would be charged if the trucks come in with a relatively low loading ratio in order to improve the utilization efficiency and to reduce relevant carbon emission.

## 7. Conclusions and Implications

Based on the big datasets, this research analyzes the freight characteristics and carbon emission of construction waste hauling trucks (CWHTs) in Hong Kong. The results reveal the following: (a) average daily CWHTs’ trip was 4544 on working days and 553 (trip to landfills accounts for the largest portion) on non-working days; (b) medium and heavy trucks are commonly used for transporting construction waste; (c) the majority of CWHTs’ trips were short and medium trips, with a distance of less than 30 km, naturally drivers are inclined to choose the nearby disposal facilities; (d) the CWHTs in Hong Kong emitted 307.64 and 28.78 tons CO_2_-eq of carbon emission separately on working days and non-working days, and trips to landfill facilities have the lowest carbon emission efficiency; (e) busy time for CWHTs’ trips and the disposal facilities are 8:00–11:00 a.m. and 1:30–4:00 a.m., and 8:30–11:00 a.m. and 1:30–5:00 p.m., respectively on working days, while there is no obvious intensive period on non-working days.

This research offers a comprehensive understanding of CWHTs freight characteristics and carbon emission performance. One implication of the research is that governments are suggested to propose a dynamic pricing system with different transporting fees in order to disperse the freight transportation of CWHTs. Higher transporting fees can be initiated for off-peak hours or non-working days in order to improve transportation efficiency; therefore, a low-load fee can be charged to deal with the low loading ratio of trucks. Another implication is that it provides important benchmarking metrics for monitoring the effectiveness of any policy interventions related to carbon emission. Freight carbon emission was found to be related to the vehicle type, transporting weight, and trip length, while trip length was the most considerable metric to carbon emission. The demands of transporting construction waste need to be monitored in order to choose suitable vehicle types and disposal facilities. It is vital to increase the loading ratio of the CWHTs and thus reduce the overall trip length. This study provides new insights for investigating freight characteristics and carbon emissions, which is significant reference material for other cities around the world.

This paper provides a springboard for further research. First, this research follows the assumption that the trip length and duration extracted are unrelated to the actual traffic situation, such as traffic congestion and infrastructure changes. Future research can make more accurate time-of-day profiles by considering the real-time traffic. Second, our research implies the potential of a dynamic pricing system to disperse the freight transportation of CWHTs, but this system is yet to be further developed by considering the demands of transporting construction waste, the price sensitivity of CWHT drivers, and many other factors. Third, the method for optimizing construction waste collection routing can be extended to further study in order to improve the efficiency of waste transportation and reduce carbon emission.

## Figures and Tables

**Table 1:** List of freight performance metrics.

Categories	Performance Metrics	Description	References
Vehicle-related	Vehicle type	Vehicles are normally classified into different types based on their load capacity as follows: light vehicles, medium vehicles, and heavy vehicles.	Combes and Leurent, 2013 [18], D’Este, 2007 [20], Khan and Machemehl, 2017 [19]
	Commodity type	The key commodities include building and construction materials, consumer goods, industrial inputs, and waste.	Beliën et al., 2014 [17], Combes and Leurent, 2013 [18], Errampalli et al., 2020 [21], Ruan et al., 2012 [22]
	Weight capacity	The weight of commodity carried for a trip.	Combes and Leurent, 2013 [18], D’Este, 2007 [20], Errampalli et al., 2020 [21]
	Permitted gross vehicle weight	The maximum permitted loading weight.	D’Este, 2007 [20], Lu, 2019 [23]
	Ownership of the vehicles	Ownership of the vehicles can be divided into private companies, government, and individuals.	Errampalli et al., 2020 [21]
Trip-related	Trip origin	Location of the trip origin.	Combes and Leurent, 2013 [18], Nuzzolo et al., 2020 [24]
	Trip destination	Location of the trip destination.	Akter, 2019 [25], Combes and Leurent, 2013 [18]
	Departure time	The time starting from the origin.	Akter, 2019 [25], Nuzzolo et al., 2020 [24], Ruan et al., 2012 [22]
	Arrival time	The time reaching the destination.	Akter, 2019 [25], Ruan et al., 2012 [22]
	Trip length	Distance traveled by the truck from the origin of the trip to the destination.	Combes and Leurent, 2013 [18], Khan and Machemehl, 2017 [19], Nuzzolo et al., 2020 [24]
	Trip time	Time taken to travel from the origin of the trip to the destination.	Akter, 2019 [25], FHWA, 2017 [26], Khan and Machemehl, 2017 [19], Nuzzolo et al., 2020 [24], Schrank et al., 2012 [27]
	Trip speed	The average speed of the trip between the origin and the destination.	FHWA, 2017 [26], Khan and Machemehl, 2017 [19]
	Number of stops	The number of stops for a trip.	Akter, 2019 [25], D’Este, 2007 [20], Nuzzolo et al., 2020 [24], Ruan et al., 2012 [22]
	Routing type	Variable, regular, fixed.	D’Este, 2007 [20]

**Table 2:** Government construction waste disposal facilities.

Facility Type	Existing Facilities	Abbreviation	Type of Accepted Construction Waste
Public fills	Chai Wan Public Fill Barging Point	CW-PFBP	Entirely inert construction waste
	Mui Wo Temporary Public Fill Reception Facility	MW–PFRF	
	Fill Bank at Tseung Kwan O Area 137	TKO137FB	
	Fill Bank at Tuen Mun Area 38	TM38–FB	
Sorting facilities	Sorting Facilities at Tseung Kwan O Area 137	TKO137SF	More than 50%, by weight, of inert construction waste
	Sorting Facilities at Tuen Mun Area 38	TM38–SF	
Landfill facilities	Northeast New Territories Landfill	NENT	Not more than 50%, by weight, of inert construction waste
	Southeast New Territories Landfill	SENT	
	West New Territories Landfill	WENT	

Source: “*Hong Kong Green Organisation Certification Guidebook*” for carbon reduction certificate. 2021. https://www.hkgoc.gov.hk/en-hk/carbon-reduction-certificate.html. Accessed on 13 February 2022.

**Table 3:** Details of construction waste disposal records.

Record	Description
Facility	The government waste disposal facilities for construction waste, as well as the destination of the construction waste hauling trucks for a trip.
Vehicle no	The license plate number of the trucks involved in transportation.
Transaction date	The date when the freight occurs.
Time-in	The time when the vehicle enters the facility, as well as the arrival time for a trip.
Time-out	The time when the vehicle exits the facility.
Net weight	The total weight of construction waste carried by hauling truck per trip.

**Table 4:** Average energy consumption based on gross vehicle weight.

Gross Vehicle Weight (GVW)	Average Energy Consumption (L/100 km)
GVW ≤ 2.5 tons	10.2
2.5 tons < GVW ≤ 4 tons	12.2
4 tons < GVW ≤ 5.5 tons	18.6
5.5 tons < GVW ≤ 10 tons	31.9
10 tons < GVW ≤ 15 tons	34.3
15 tons < GVW ≤ 20 tons	44.3
20 tons < GVW ≤ 24 tons	54.1
24 tons < GVW ≤ 38 tons	61.1

Data source: https://www.cleanair.hk/eng/index.htm, accessed on 30 April 2021.

**Table 5:** Detailed parameters for calculating carbon emissions.

Vehicle Type	CO_2_		CH_4_		NO_2_		Transferring Factor
	Emission Factor (kg/L)	GWP	Emission Factor (kg/L)	GWP	Emission Factor (kg/L)	GWP	
Light CWHTs	2.614	1	0.072 × 10^−3^	21	0.506 × 10^−3^	310	2.78
Medium CWTHs			0.145 × 10^−3^		0.072 × 10^−3^		2.84
Heavy CWHTs			0.145 × 10^−4^		0.072 × 10^−4^		2.84

**Table 6:** Distribution of trips by vehicle types.

Freight Trip Type	Facility	Light Truck (0 < PGVW ≤ 5.5)	Medium Truck (5.5 < PGVW ≤ 24)	Heavy Truck (24 < PGVW ≤ 38)	Total Trips
**TLF**	**NENT**	14 (0.28%)	4468 (87.97%)	597 (11.75%)	5079
	**SENT**	76 (0.61%)	9061 (72.87%)	3297 (26.52%)	12,434
	**WENT**	5 (0.20%)	1174 (45.84%)	1382 (53.96%)	2561
**TSF**	**TKO137SF**	217 (3.28%)	6023 (90.97%)	381 (5.75%)	6621
	**TM38–SF**	24 (0.70%)	2890 (83.72%)	538 (15.59%)	3452
**TPF**	**MW–PFRF**	5 (0.42%)	1198 (99.58%)		1203
	**TKO137FB**	14 (0.03%)	16,427 (37.29%)	27,612 (62.68%)	44,053
	**TM38–FB**	1 (0.02%)	8042 (31.00%)	29,496 (68.98%)	37,539
**Total trips**		356 (0.32%)	49,283 (43.64%)	63,303 (56.05%)	112,942

TLF denotes the trips to landfill facilities; TPF denotes trips to public fills; TSF denotes trips to sorting facilities.

**Table 7:** Carbon emission efficiency of construction waste transportation.

Trip Type		TLF	TSF	TPF
**Total transporting weight (ton)**	a	100,694	54,504	1153,40
**Total carbon emission (kg)**	b	1,264,269	467,825	5,852,637
**Carbon emission efficiency of construction waste transportation (kg CO_2_-eq/ton waste)**	b/a	12.56	8.58	5.07

Notes: TLF = trips to landfill facilities, TSF = trips to sorting facilities, and TPF = trips to public fills.

**Table 8:** Daily freight performance and carbon emission.

	Travel Types	TLF	TSF	TPF	Daily
**Working days**	**Number of trips**	763 (17%)	392 (9%)	3390 (74%)	4544
	**Transporting weight (ton)**	3704.06 (7%)	2135.67 (4%)	47,270.79 (89%)	53,110.52
	**Trip length (km)**	19,488.03 (17%)	7711.85 (6%)	89,510.99 (77%)	116,711.86
	**Trip duration (h)**	565.89 (20%)	182.40 (7%)	1997.82 (73%)	2746.11
	**Carbon emission (ton CO_2_-eq)**	48.45 (16%)	18.31 (6%)	240.87 (78%)	307.64
	**Trip duration/Trip length (h/100 km)**	2.90	2.37	2.23	2.35
**Non-working days**	**Number of trips**	252 (46%)	96 (17%)	205 (37%)	553
	**Transporting weight (ton)**	1685.34 (33%)	464.12 (9%)	2977.28 (58%)	5126.75
	**Trip length (km)**	5688.42 (50%)	1861.84 (16%)	3791.98 (34%)	11,342.24
	**Trip duration (h)**	176.35 (58%)	43.93 (14%)	85.91 (28%)	306.18
	**Carbon emission (ton CO_2_-eq)**	14.49 (50%)	4.05 (14%)	10.24 (36%)	28.78
	**Trip duration/Trip length (h/100 km)**	3.10	2.36	2.27	2.69

Notes: TLF represents trips to landfill facilities, TSF represents trips to sorting facilities, and TPF represents trips to public fills.

## Data Availability

Not applicable.
